# Complex Analysis of Endothelial Markers as Potential Prognostic Indicators in Luminal Invasive Breast Carcinoma Patients: Outcomes of a Six-Year Observational Study

**DOI:** 10.3390/biomedicines11082246

**Published:** 2023-08-10

**Authors:** Katarzyna Kwiatkowska, Piotr Rhone, Paulina Koziorzemska, Dorota Formanowicz, Barbara Ruszkowska-Ciastek

**Affiliations:** 1 Department of Pathophysiology, Faculty of Pharmacy, Nicolaus Copernicus University, Collegium Medicum, 85-094 Bydgoszcz, Poland; koziorzemskap@gmail.com; 2 Clinical Ward of Breast Cancer and Reconstructive Surgery, Oncology Centre Prof. F. Łukaszczyk Memorial Hospital, 85-796 Bydgoszcz, Poland; rhonep@co.bydgoszcz.pl; 3 Department of Medical Chemistry and Laboratory Medicine, Poznan University of Medical Sciences, 60-806 Poznan, Poland; doforman@ump.edu.pl; 4 Department of Stem Cells and Regenerative Medicine, Institute of Natural Fibres and Medicinal Plants-National Research, 62-064 Plewiska, Poland

**Keywords:** breast cancer, LAR, sP-selectin, sE-selectin, von Willebrand factor, relapse, treatment

## Abstract

(1) Background: Metastasis is a complex process in which the primary cancer cells spread to a distant organ or organs, creating a secondary tumor location, which in many patients leads to treatment failure and death. The aim of the present study was to assess the association of endothelial markers (i.e., sP-selectin, sE-selectin and von Willebrand factor) with the leptin-to-adiponectin ratio (LAR) and to perform an analysis of the predictive value on the survival of patients with luminal A and B invasive breast cancer (IBrC). (2) Methods: The trial included 70 treatment-naïve early-stage IBrC patients with a median age of 54.5 years and a median tumor diameter of 1.5 cm. The median duration of follow-up was 5.7 years, with a relapse rate of 15.71%. Specific immunoenzymatic kits were used to determine pre- and post-treatment concentrations of analyzed factors. (3) Results: Regardless of the treatment pattern, endothelial marker concentrations and the LAR increased after adjuvant treatment. The follow-up showed a significantly higher relapse rate in patients with IBrC who had higher pre-treatment sP-selectin and post-treatment LAR levels. According to receiver operating characteristic (ROC) analysis, a post-treatment LAR with a sensitivity of 88.9% and specificity of 57.9% discriminating cases with or without disease relapse. Additionally, a higher risk of breast cancer relapse was associated with a lower post-treatment sP-selectin concentration. (4) Conclusions: Our results showed mainly that pre-treatment sP-selectin levels and post-treatment LAR may have value as prognostic indicators and may contribute to predicting the future outcomes in patients with early-stage IBrC.

## 1. Introduction

Metastasis is a multi-step process by which primary tumor cells migrate to a distant organ(s) in order to create a secondary tumor site. It is a characteristic of cancer that leads to treatment failure and the death of many patients [1]. As a result, the prognosis of the patient is closely related to metastasis. At diagnosis, 5–10% of breast cancer patients have metastases, and 30–40% of women with early breast cancer develop metastases during the disease [2,3]. Breast cancer is a clinically, pathologically, histologically, and prognostically complex disease and the classification that is important to determine treatment and the future outcome is based on analysis of the estrogen receptor (ER), the progesterone receptor (PR), human epidermal growth factor receptor 2 (HER2), and proliferation marker (Ki67). Estimation of these molecular indicators enables distinguishing four general intrinsic breast cancer subtypes: luminal A, luminal B (hormonal receptor positive), non-luminal HER2 positive, and triple negative. These subtypes have a wide range of metastases, prognoses, and treatment options [4]. Different gene and protein expression profiles are likely to explain the different patterns of metastasis and survival in different breast cancer subtypes [5].

Adipose tissue is now considered to be one of the largest endocrine organs, which secretes dozens of adipokines, including leptin (pro-inflammatory), adiponectin (anti-inflammatory), resistin, interleukin-1 (IL-1), and interleukin-6 (IL-6) [6]. By excessive release of free fatty acids, tumor necrosis factor α (TNF-α), IL-1, IL-6, and other mediators regulate endothelial function and induce endothelial damage. Thus, adipocytes are regarded as a significant source of chronic low-grade inflammation. Endothelial cells release excessive amounts of cell adhesion molecules (CAMs), such as vascular cell adhesion molecule-1 (VCAM-1), intercellular adhesion molecule-1 (ICAM-1), and E-selectin in response to subclinical inflammation, resulting in leukocyte mobilization and adherence to the endothelium [7].

CAMs are a type of protein that play an important role in the motility, differentiation, proliferation, migration, and apoptosis of primary tumor cells and intravasation through the endothelium into blood vessels during the metastatic process of cancer. CAMs are responsible for maintaining tissue continuity under physiological conditions by interacting with cells and the extracellular matrix [8,9,10]. Impaired adhesion molecule function at any stage can contribute to the loss of normal cell–cell interactions, allowing cancer cells to dedifferentiate and spread [8]. The most recognized and studied CAMs include selectins. Leukocyte-selectin (L-selectin, CD62L), endothelial-selectin (E-selectin, CD62E) and platelet-selectin (P-selectin, CD62P) are the three selectin subfamily members [11]. The essential role of selectins is facilitation of leukocyte adhesion and rolling on the vessel wall surface in the inflammatory milieu. However, selectin-dependent tumor cell spread is linked with attachment and diapedesis of tumor cells through the endothelium and formation of a metastatic niche [12,13].

The appearance of E-selectin ligands on cancer cells, for example, mucins, dead receptor-3 and a specific CD44 glycoform, is linked to increased adhesion to activated endothelial cells. The binding of E-selectin to death receptor 3 or other ligands on cancer cells has been shown to improve survival during metastasis [14,15]. Soluble platelet-selectin (sP-selectin) is a soluble form of P-selectin that is released by granules and is detectable in plasma [16]. P-selectin, which allows tumor cell aggregates to adhere to the endothelium and then extravasate, is one of the proteins that mediates the close interaction between platelets and tumor cells [17,18]. However, the most powerful endothelial activation marker is von Willebrand factor (vWF). Despite its role in platelets adhesion and secondary coagulation, it contributes to cancer growth and dissemination [19]. Disease progression and worse future prognosis were associated with high vWF levels by Dhami et al., who suggest that the vWF concentration may serve as an independent prognostic marker in neoplastic disease [20]. Rhone et al. noted opposite results since a lower concentration of vWF was related to a shorter survival rate [21]. Discrepancies in this regard might be associated with an unrevealed role of vWF in cancerogenesis.

Interestingly, leptin and adiponectin are the major adipokines, which present opposite properties in metabolism, immune response, reproductive process and cancerogenesis. A high concentration of leptin promotes cancer cell proliferation and diminishes its apoptosis [22]. Numerous hormone-dependent cancers are associated with a lower adiponectin concentration, i.e., breast, endometrial, and prostate [22,23,24]. Thus, the leptin-to-adiponectin ratio (LAR) was established as a sensitive systemic inflammatory marker and predictor of cardiometabolic and neoplastic disease outcomes [7]. Since, Słomian et al. observed that lower LAR was associated with a longer survival rate and better therapy response. Additionally, adiponectin and leptin investigated separately do not correspond to the stage of ovarian cancer and response to chemotherapy [25]. Iwan-Zietek et al. have found an inverse association between the LAR and soluble form of P-selectin in morbidly obese patients, which may suggest reduced platelet aggregation [26]. Thus, the aim of the present study was to assess the effect of endothelial markers before and after treatment (i.e., sP-selectin, sE-selectin and vWF) on the LAR (also assessed before and after treatment) and perform an analysis of the predictive value of these parameters on the survival of patients with luminal A and B invasive breast cancer (IBrC). To determine the accuracy of our predictive model, we used Kaplan–Meier analysis, linear regression, and the receiver operating characteristic (ROC) curve in this study.

## 2. Materials and Methods

### 2.1. Patient Enrolment

**Figure 1:**
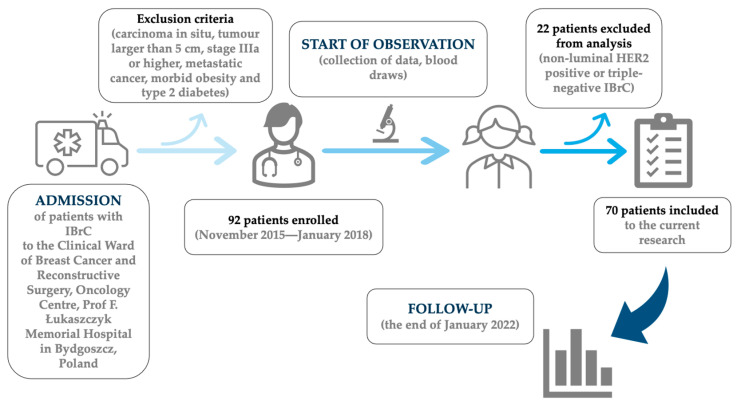
Flowchart of the current research.

This study included 70 previously untreated patients with clinically and histologically proven primary, invasive, unilateral, non-metastatic, early-stage (IA–IIB) IBrC. The flowchart of patients enrolled for this study is shown in Figure 1.

### 2.2. Tumor Characteristics

Comprehensive patient clinico-pathological characteristics are presented in Table 1. All patients showed positive estrogen receptor status and only 5 women demonstrated progesterone receptor-negative staining. Fifty patients demonstrated lower than 20% expression of Ki-67. Invasive ductal carcinoma was detected in 61 (87%) women. Tumor diameter lower than 2 cm was exhibited in 48 cases (69%). Seventeen patients demonstrated lymph node metastasis. Based on these facts, 50% (35 cases) of the study group had stage I IBrC.

### 2.3. Follow-Up

Patients were followed from the date of IBrC diagnosis until the date of breast cancer recurrence or death or until January 2022, whichever came first. The period from study inclusion to the date of recurrence is described as disease-free survival (DFS), and the time to the patient’s last visit or death is defined as overall survival (OS). The median follow-up was 68.5 months (IQR = 59–72 months). There were 11 events during this study, including one distant metastasis and ten deaths (recurrence rate: 15.71%).

### 2.4. Ethics Statement

This study was performed under the appropriate institutional ethics approvals (KB 547/2015) and in accordance with the guidelines of the Declaration of Helsinki. Written informed consent was obtain from each participant.

### 2.5. Treatment Requirements

All individuals were treated in accordance with the National Comprehensive Cancer Network (NCCN) Guidelines for Practice. Fifty-six patients received breast-conserving surgery (BCS), seven had a conventional mastectomy, and seven underwent a modified radical mastectomy (MRM). All surgical operations were performed under normal procedures. Adjuvant therapy was given to 68 women. Surgery was the initial treatment for all research participants, followed by adjuvant therapy that included radiation, brachytherapy, hormone therapy, chemotherapy, and immunotherapy. Post-operative radiation was mostly given to patients who had undergone BCS. In the study group, post-operative radiation was delivered in 17–20 fractions over 4–6 weeks using X photons with energy of 6/15 MeV and a dosage of 42.5 gray (Gy). Moreover, brachytherapy at a dosage of 10 Gy was administered to the tumor bed in half of the women. Adjuvant chemotherapies included anthracycline-containing (*n* = 23) and non-anthracycline-containing (*n* = 4) medicines administered in three to six cycles. Menopausal status determined the type of endocrine treatment; 40 (57%) received tamoxifen (Egis Pharmaceuticals, Budapest, Hungary), 17 (24%) received aromatase inhibitors (AIs) (Arimidex [anastrozole], AstraZeneca, Cambridge, UK), and 7 (10%) received a combination of tamoxifen and AIs. Adjuvant immunotherapy was necessary for four HER2-positive individuals (6%) (trastuzumab).

### 2.6. Sample Collection and Analysis

Venous blood samples were taken twice into 4.5 mL tubes (BD Vacutainer^®^ Plus Plastic Serum Tubes, Franklin Lakes, NJ, USA) without anticoagulant to determine adiponectin, leptin, and soluble forms of E-selectin (sE-selectin) and P-selectin (sP-selectin) concentrations. In tubes (BD^TM^ Vacutainer^TM^ Citrate Tube) containing an anticoagulant additive equivalent to 3.2% trisodium citrate for coagulation investigations, 4.5 mL for von Willebrand factor analysis were collected. Material was gathered under strict condition. Samples taken for lab analyses were confined to one freeze–thaw cycle.

The first blood sample was taken 24 h before the surgical procedure (I—pre-treatment values). In order to minimize effects of adjuvant treatment, the subsequent blood specimen (II—post-treatment) was collected nine months (IQR = 6.0–10.0) after the cancer surgery.

#### 2.6.1. Leptin-to-Adiponectin Ratio

The Human Leptin Enzyme-Linked Immunosorbent Assay (ELISA) Clinical Range (BioVendor Research and Diagnostic Products, Brno, Czech Republic; catalogue number: RD191001100) were used to measure baseline serum pre- and post-treatment leptin concentrations. The detection limit for leptin was 0.2 ng/mL. The intra-assay coefficient of variation (within run) was 5.9%, with a run-to-run coefficient of variation of 5.6%.

A human adiponectin ELISA high-sensitivity ELISA kit was used to measure pre- and post-treatment serum adiponectin levels (BioVendor Research and Diagnostic Products, Brno, Czech Republic; catalogue number: RD191023100). The detection limit for adiponectin was 0.47 ng/mL, with a 3.9% intra-assay coefficient of variation (within-run) and a 6.0% inter-assay coefficient of variation (run-to-run).

The leptin-to-adiponectin ratio was assessed using the following formula:

$$LAR=\frac{leptin}{adiponectin}.$$

#### 2.6.2. Serum sE-Selectin Measurements

Serum pre- and post-treatment sE-selectin level were determined by the Diaclone CD62E/ELAM-1 ELISA Set (Diaclone SAS, Besancon Cedex, France; catalogue number: 851.580). The detection limit was 0.5 ng/mL, with an assay range of 1 ng/mL to 32 ng/mL.

#### 2.6.3. Serum sP-Selectin Analysis

Serum pre- and post- treatment sP-selectin level were measured using a commercially available kit, Enzyme-linked Immunosorbent Assay (ELISA) Kit for sP-selectin (SELP) (Cloud-Clone Corp., Katy, TX, USA, catalogue number: SEA569Hu). The sP-selectin detection limit was 27 pg/mL. The intra-assay coefficient of variation (within-run) was 10%, with an inter-assay coefficient of variation (run-to-run) of 12%.

#### 2.6.4. Von Willebrand Factor Antigen Measurements

Plasma pre- and post-treatment concentrations of von Willebrand factor were determined using the Imubind^®^ vWF ELISA ref: 828 (BioMedica Diagnostics, Stamford, CT, USA) test. The vWF detection limit was 0.1 mU/mL, with an assay range of 0–10 mU/mL.

#### 2.6.5. Immunohistochemistry (IHC) Analysis

The evaluation of ER and PR status, expression of HER2, and Ki67 was done using IHC. ER and PR status were evaluated using SP1 and 1E2 primary antibodies (Ventana Medical Systems, Tucson, AZ, USA) in line with ASCO and CAP standards. Hormone receptor status was characterized as positive if there was at least 1% of tumor cells with nuclear staining and negative if there was no nuclear staining at all. The rabbit monoclonal primary antibody VENTANA anti-HER2/neu (4B5) was used with a VENTANA aperture to stain the IHC microscope slide (Benchmark Ultra, Roche-Ventana) for semi-quantitative identification of HER2. On a scale of 0, 1+, 2+, and 3+, HER2 scores were calculated using the usual ASCO/CAP guideline reporting method. Tumors with a score of 0 or 1+ were classified as HER2 negative, whereas those with a value of 3+ were labelled HER2 positive. Tumors with 2+ scores were deemed ambiguous and subjected to fluorescence in situ hybridization (FISH) with a dual HER2/Cep17 probe. Using a monoclonal mouse antibody (Auto-stainer Link 48, Agilent Technologies, Santa Clara, CA, USA), the Ki67 antigen was scored as a percentage of nuclei-stained cells in all cancer cells. We utilized a 20% threshold to designate high or low proliferative instances in the Ki67 proliferation index.

### 2.7. Statistical Analysis

Statistica version 13.1 (StatStoft^®^, Cracow, Poland) was used for statistical analysis. The Shapiro–Wilk test was used to ensure that the data distribution was normal. Student’s *t*-test (normal distribution) or the Mann–Whitney U test was used to compare two groups of continuous data (non-normal distribution). Univariate ANOVA analysis with normal distributions or the Kruskal–Wallis ANOVA analysis with non-normal distributions was used to compare more than two groups of continuous data. As appropriate, patient data are presented as the mean and standard deviation or median and interquartile range (IQR). In brackets ‘()’, we have given the standard deviation and the values separated by a slash ‘/’ are Q1 and Q3. In addition, the data for two dependent variables were compared using a non-parametric Wilcoxon signed rank test. The relationships between the parameters under investigation were examined using Spearman’s rank order correlation test. The investigation also included the use of ROC curves, AUC (area under a curve), and Youden’s index (see Supplementary Materials). Cut-off values were determined based on the ROC and median. Survival times were expressed using Kaplan–Meier curves, and the log-rank test was utilized to compare survival times (statistically non-significant results have been moved in the Supplementary Materials). The term OS refers to the time between the start of randomization or treatment and death. DFS refers to the interval between randomization or the start of a treatment and the occurrence of disease progression or death. The link between two or more independent variables and one dependent variable was estimated using multiple linear regression. The Cox proportional hazards model was used for multivariate and univariate regression analysis. To assess the independent impact of selected factors at the time of diagnosis on breast cancer survival, a multivariate Cox regression model included all variables with a significant effect in the univariate analysis. All analyses performed were summarized and reported in tables and figures. The statistical significance cut-off value was set at a *p*-value < 0.05.

## 3. Results

### 3.1. Baseline Characteristics

We identified seventy women with non-metastatic (M0), early-stage (stage I–II) invasive breast cancer. Table 1 and Figure 1 demonstrate baseline patients characteristics. Median (IQR—interquartile range) age at cancer diagnosis in the overall cohort was 54.5 (49.0–59.0) years. There were 26 premenopausal women and 44 postmenopausal women among the 70 women. There were 48 T1 patients and 22 T2 patients in the TNM classification of breast cancer. The median (IQR) tumor diameter was 1.5 (1.1–2.1) cm. BCS was performed on 56 patients, and 14 had a mastectomy. Adjuvant chemotherapy was administered in 27 patients and 2 women did not require endocrine treatment. All patients were identified to explore the prognostics and future outcomes.

### 3.2. LAR Levels Prior to and after Treatment in Relation to the Types of Therapy

Table 2 presents the LAR regarding the types of therapy. Regardless of the treatment pattern, the LAR increased after treatment.

### 3.3. Patients’ Treatment in Relation to Their Pre- and Post-Treatment sE-Selectin Concentrations

Table 3 presents the sE-selectin concentrations related to treatment strategy. Regardless of the treatment pattern, the sE-selectin concentration increased after treatment. The pre-treatment sE-selectin concentrations were higher in patients who received breast-conserving therapy with a trend towards statistical significance (*p* = 0.0840). Considering the types of chemotherapy, pre-treatment sE-selectin concentrations were higher in patients treated with non-anthracycline chemotherapy (*p* = 0.0125). Surprisingly, post-treatment sE-selectin concentrations were higher in patients who had not been treated with chemotherapy (*p* = 0.0081).

### 3.4. Relationship between sP-Selectin Concentrations before and after Treatment

Table 4 presents the sP-selectin concentrations with regard to the types of therapy. Regardless of the treatment pattern, the sP-selectin concentration increased after treatment. Considering the types of endocrine therapy, post-treatment sP-selectin concentrations were higher in patients who had not been treated with endocrine therapy (*p* = 0.0015), but this observation needs to be confirmed in a group with larger numbers.

### 3.5. vWF Concentrations Prior to and after Treatment in Relation to the Types of Therapy

Table 5 shows the vWF concentrations in relation to therapy type. The vWF concentration increased after treatment regardless of treatment pattern. Post-treatment vWF concentrations were higher in patients who received chemotherapy based on anthracycline (*p* = 0.0486).

### 3.6. Association between Endothelial Markers and Pre-Treatment LAR Values

The next step in the statistical analysis (Table 6) was to test the pre-treatment and post-treatment concentrations of sE-selectin, sP-selectin and vWF against the pre-treatment LAR value. We divided the breast cancer patients into three subgroups—those with low (<0.27), moderate (0.27–0.65) and high (>0.65) pre-treatment LAR values. No statistically significant data were observed in this analysis.

### 3.7. Association between Endothelial Markers and Post-Treatment LAR Values

The pre-treatment and post-treatment concentrations of sE-selectin, sP-selectin, and vWF were then tested against the post-treatment value of the LAR in the statistical analysis (Table 7). We divided the breast cancer patients into three subgroups based on post-treatment LAR values: low (<0.60), moderate (0.60–1.04), and high (>1.04). With a trend towards statistical significance, the higher the post-treatment LAR level in breast cancer patients, the higher the pre-treatment sP-selectin as a result (*p* = 0.0528).

### 3.8. Correlation Analysis of Clinical Parameters before and after Treatment

**Figure 2:**
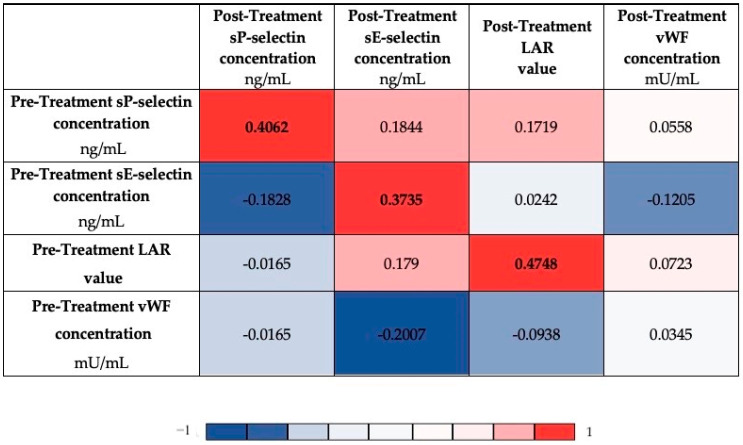
Heatmap displaying the r values obtained from Spearman’s correlation analysis performed among investigated markers; *p*-values < 0.05 were considered to indicate statistical significance and are marked in bold.

Figure 2 shows the correlation analysis that was performed to find the relationship between LAR and endothelial markers before and after treatment. The analysis was performed using Spearman’s rank correlation and is presented in the form of heatmap. As a result, the pre-treatment markers (sP-selectin, sE-selectin, and LAR) were found to correlate positively with their post-treatment counterparts (r = 0.4062, r = 0.3735 and 0.4748, respectively), apart from vWF concentrations before and after treatment.

### 3.9. Association of the Analysed Parameters with DFS in Linear Regression

The next step in the statistical analysis (Table 8) was to determine the associations of pre- and post-treatment LAR and endothelial markers and DFS by multiple linear regression. A higher risk of breast cancer relapse was associated with a lower post-treatment sP-selectin concentration (Beta = −0.2576, *p* = 0.0504) detected by linear regression (Model 3). Similarly, in Model 4, adjusted for age, BMI, parity, menopausal status, smoking status, tumor stage, tumor diameter, intrinsic type, histological type, and nodal involvement, the outcome demonstrated a tendency towards significance, with a higher risk of breast cancer relapse associated with a lower post-treatment sP-selectin concentration (Beta = −0.2437, *p* = 0.0583).

### 3.10. Survival Analysis Regarding Pre- and Post-Treatment LAR and Endothelial Markers

In the statistical analysis, the cut-off point based on the median were determined using the cut-off points from the ROC curve for the LAR and endothelial markers before and after treatment (Tables S2 and S3 in the Supplementary Materials). The cut-off points divided patients into 2 subgroups: those with above and below the cut-off points. During 68.5 months of follow-up we found 10 cancer-related deaths. One patient only relapsed. The relapse rate was 15.71%. Subsequently, we performed Kaplan–Meier curves in order to determine OS and DFS of each group. Those that are not statistically significant were transferred to the Supplementary Materials (pre-treatment LAR value (Figure S1), pre-treatment sE-selectin concentration (Figure S2), post-treatment sE-selectin concentration (Figure S3), post-treatment vWF concentration (Figure S4)).

**Figure 3:**
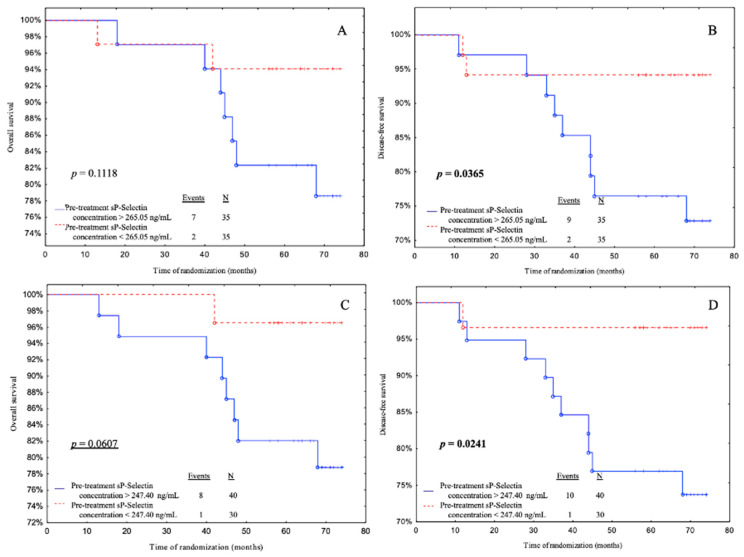
Overall survival (OS) and disease-free survival (DFS) for pre-treatment sP-selectin concentrations in cohort. Kaplan–Meier plots illustrating patients survival based on the OS and DFS regarding (**A**,**B**) median value cut-off and (**C**,**D**) ROC cut-off. *p*-values < 0.05 were considered to indicate statistical significance and are marked in bold, underlined *p*-values represent closeness to statistical significance.

Subjects with pre-treatment sP-selectin levels less than 265.05 ng/mL had a significantly better DFS than patients with pre-treatment sP-selectin levels greater than 265.05 ng/mL according to median value cut-off (*p* = 0.0365) (Figure 3B). Additionally, patients with pre-treatment sP-selectin levels below 247.40 ng/mL (ROC cut-off) had a better OS (with a tendency to significance *p* = 0.0607) and DFS than patients with pre-treatment sP-selectin levels above 247.40 ng/mL (*p* = 0.0241) (Figure 3C,D).

**Figure 4:**
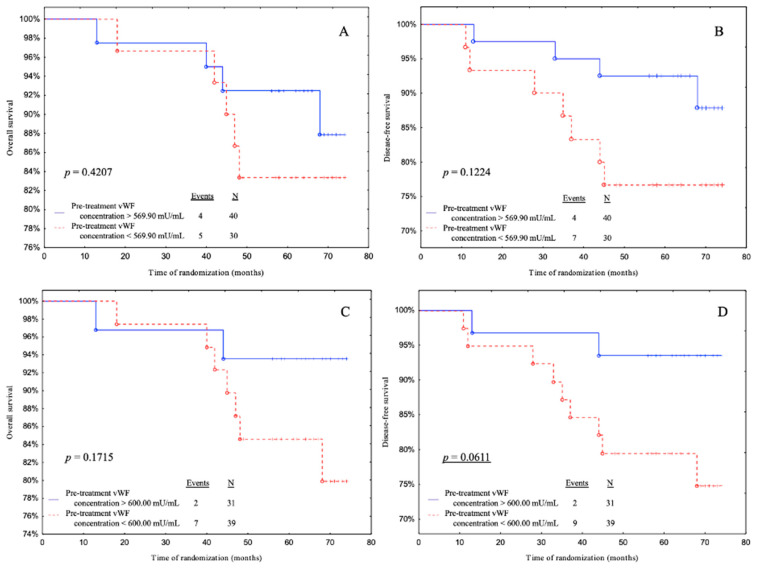
Overall survival (OS) and disease-free survival (DFS) for pre-treatment vWF concentrations in cohort. Kaplan–Meier plots illustrating patients survival based on the OS and DFS regarding (**A**,**B**) median value cut-off and (**C**,**D**) ROC cut-off. Underlined *p*-values represent closeness to statistical significance.

Subjects with pre-treatment vWF levels above 600.00 mU/mL (ROC cut-off) had a significantly better DFS than patients with pre-treatment vWF levels below 600.00 mU/mL with a tendency to significance (*p* = 0.0611) (Figure 4D).

**Figure 5:**
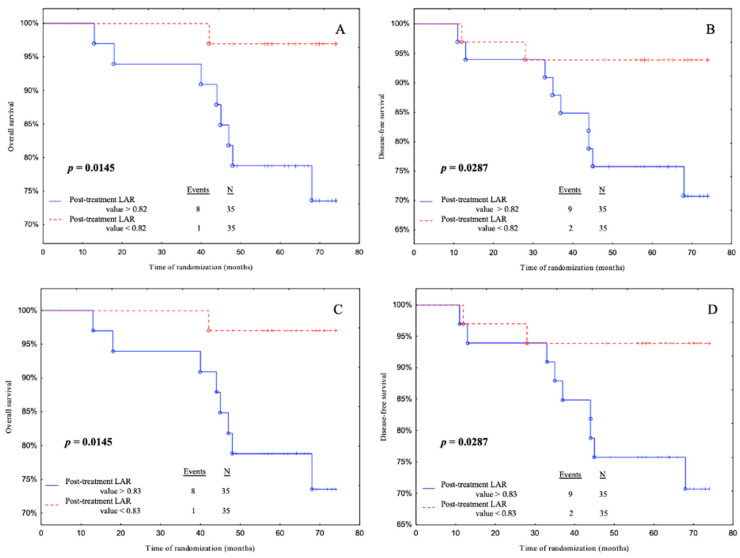
Overall survival (OS) and disease-free survival (DFS) for post-treatment LAR values in cohort. Kaplan–Meier plots illustrating patients survival based on the OS and DFS regarding (**A**,**B**) median value cut-off and (**C**,**D**) ROC cut-off. *p*-values < 0.05 were considered to indicate statistical significance and are marked in bold, underlined *p*-values represent closeness to statistical significance.

Subjects with post-treatment LAR levels below 0.82 had a significantly better OS and DFS than patients with post-treatment LAR levels above 0.82 according to median cut-off (*p* = 0.0145, *p* = 0.0287, respectively) (Figure 5A,B). Additionally, patients with post-treatment LAR levels below 0.83 had a better OS and DFS than patients with post-treatment LAR levels above 0.83 according to the ROC cut-off (*p* = 0.0145, *p* = 0.0287, respectively) (Figure 5C,D).

**Figure 6:**
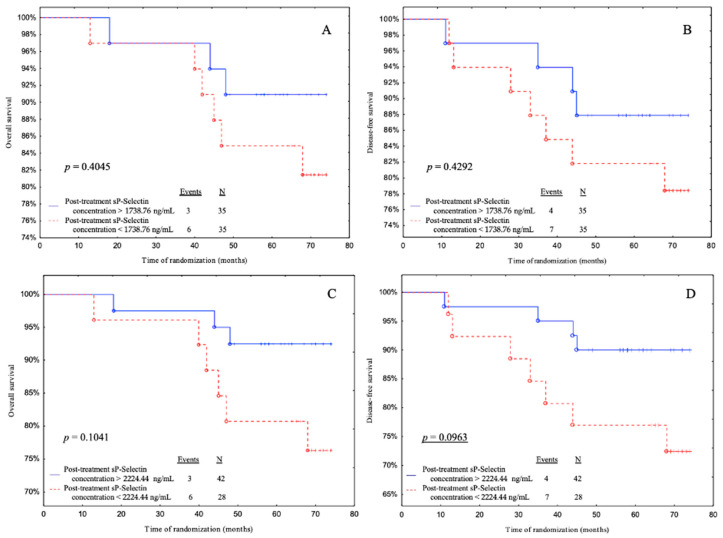
Overall survival (OS) and disease-free survival (DFS) for post-treatment sP-selectin concentrations in cohort. Kaplan–Meier plots illustrating patients survival based on the OS and DFS regarding (**A**,**B**) median value cut-off and (**C**,**D**) ROC cut-off. Underlined *p*-values represent closeness to statistical significance.

Subjects with post-treatment sP-selectin levels above 2224.44 ng/mL (ROC cut-off) had a significantly better DFS than patients with post-treatment sP-selectin levels below 2224.44 ng/mL with a tendency to significance (*p* = 0.0963) (Figure 6D).

### 3.11. Univariate and Multivariate Cox Proportional Hazards Regression Models Applied to Determine Prognostic Values of Tested Parameters

The results of hazard ratio and confidence interval were provided by Cox univariate and multivariate regression models are shown in Table 9. BMI, age at diagnosis, smoking status, tumor stage, intrinsic type, histological type, nodal involvement, and tumor diameter were among the prognostic characteristics that were considered when creating the multivariate Cox regression model.

In the adjusted logistic regression analysis reporting an increase in the risk of disease relapse with an increase in the pre-treatment LAR value according to the median, also post-treatment LAR value according to both cut-off points (*p* = 0.0371; *p* = 0.0274, respectively) for DFS demonstrated prognostic values. According to the cut-off point from the ROC curve a higher post-treatment vWF concentration was associated with increase in the risk of relapse (*p* = 0.0681). However, the concentration of pre-treatment sE-selectin and vWF concentration with ROC cut-off indicated opposite associations for DFS (*p* = 0.0334; *p* = 0.0880, respectively).

The univariate Cox regression model confirmed similar associations as in multivariate analysis in respect to post-treatment LAR value and pre-treatment vWF concentration for both cases according to ROC cut-off points. In addition, as the pre-treatment sP-selectin concentration increased there was an increase in the risk of disease recurrence (*p* = 0.0555; *p* = 0.0543, respectively).

**Table 9:** Disease-free survival probability estimated by multivariate and univariate Cox regression models.

Variable		Multivariate		Univariate	
		HR (95% CI)	*p*-Value	HR (95% CI)	*p*-Value
Pre-Treatment LAR valueLowHigh	Medians	11.32	**0.0371**	1.09	0.8838
		(1.16–110.81)		(0.33–3.58)	
	ROC cut-off points	2.28	0.4423	1.11	0.8643
		(0.28–18.80)		(0.33–3.81)	
Post-Treatment LAR valueLowHigh	Medians	10.32	**0.0274**	4.7	**0.0477**
		(1.30–82.12)		(1.02–21.78)	
	ROC cut-off points	10.32	**0.0274**	4.7	**0.0477**
		(1.30–82.12)		(1.02–21.78)	
Pre-Treatment sE-Selectin ConcentrationLowHigh	Medians	0.52	0.3958	0.55	0.3377
		(0.12–2.33)		(0.16–1.87)	
	ROC cut-off points	0.18	**0.0334**	0.39	0.1376
		(0.04–0.88)		(0.12–1.35)	
Post-Treatment sE-Selectin ConcentrationLowHigh	Medians	0.99	0.9849	0.81	0.7233
		(0.25–3.87)		(0.25–2.65)	
	ROC cut-off points	0.99	0.9849	0.81	0.7233
		(0.25–3.87)		(0.25–2.65)	
Pre-Treatment sP-Selectin ConcentrationLowHigh	Medians	3.31	0.1808	4.48	0.0555
		(0.57–19.10)		(0.97–20.82)	
	ROC cut-off points	5.92	0.1162	7.55	0.0543
		(0.64–54.48)		(0.96–59.19)	
Post-Treatment sP-Selectin ConcentrationLowHigh	Medians	0.58	0.5649	1.66	0.4257
		(0.09–3.65)		(0.47–5.77)	
	ROC cut-off points	2.81	0.1948	2.75	0.1081
		(0.59–13.41)		(0.80–9.48)	
Pre-Treatment vWF ConcentrationLowHigh	Medians	0.43	0.2115	0.39	0.1343
		(0.11–1.62)		(0.11–1.34)	
	ROC cut-off points	0.22	0.088	0.26	0.0841
		(0.04–1.26)		(0.06–1.20)	
Post-Treatment vWF ConcentrationLowHigh	Medians	2.58	0.2546	1.82	0.3396
		(0.51–13.17)		(0.53–6.22)	
	ROC cut-off points	6.26	0.0681	2.16	0.2029
		(0.87–44.94)		(0.66–7.09)	

Multivariate analyses were adjusted to BMI, age at the time of diagnosis, smoking status, staging, molecular type, histological type, nodal metastasis, and tumor size; significant values are presented by bold *p*-values, underlined *p*-values represent closeness to significance.

## 4. Discussion

Breast cancer poses a danger to the health of women worldwide. In 2020, there were approximately 2.26 million new cases and 680,000 new deaths from breast cancer worldwide [27]. Metastasis is a highly complex process that involves multiple cellular mechanisms such as tumor division, invasion, immune evasion, and tissue microenvironment regulation [28]. Despite recent advances in medicine, metastasis is still the leading cause of death in breast cancer patients [29].

### 4.1. Endothelial Markers before and after Treatment Depending on the Type of Treatment

In the first stage of our study, we compared endothelial markers with different types of treatment. In our investigation, the LAR value increased after treatment despite the treatment pattern. Spearman’s correlation analysis confirms that the pre-treatment LAR value correlates positively with their post-treatment substitute. Our results seem to support those of Słomian et al. who noted in patients with ovarian cancer increased LAR level after chemotherapy. Authors suggest that the LAR may serve as a predictor of the therapeutic response on anticancer drugs [25]. Sun et al. who observed a raised concentration of leptin and adiponectin in the bone marrow of patients with acute leukemia after treatment with dexamethasone. They reasoned that hypothalamic leptin resistance should be considered in obese patients with acute lymphocytic leukemia (ALL) [30]. Increased leptin levels or leptin resistance seem to be negative factors because it is believed to have pro-pro-oncogenic, proliferative, pro-angiogenic, pro-mitogenic properties [31]. The excess amount of leptin and suppression of adiponectin are predominantly associated with disease progression or a shorter survival rate [32]. Siemińska et al. found that in the more advanced prostate cancer subgroup, patients had a higher LAR value. Authors suggest that LAR express more specific indicator of adipose tissue impairment that adiponectin and leptin identified separately [33]. Our investigation group consisted of patients with estrogen receptor (ER)-positive cancers (luminal), who may benefit from endocrine therapies. Morad et al. observed a decrease in LAR in postmenopausal women treated by tamoxifen, which is inconsistent with our study. They also noted that the LAR was higher in breast tumors than in the adjacent tissue. Authors claim that estrogen exposure increased extracellular leptin expression and the LAR in vivo [34].

Furthermore, in our research we observed that pre-treatment sE-selectin concentrations were higher in patients with breast-conserving therapy (with a tendency to significance) and treated with non-anthracycline chemotherapy. Kang et al. noted that a higher sE-selectin concentration was relate positively with tumor size, grade, stage, molecular subtype, and worse future outcomes in breast cancer patients [35]. Interestingly, post-treatment sE-selectin concentrations were higher regardless of the treatment pattern and in patients not receiving chemotherapy. Ramcharan et al. observed lower sE-selectin level after 3 months in the colorectal cancer patients treated with surgery alone. However, in the group treated by surgery followed by standard chemotherapy they reported lower sE-selectin at 6 months compared with baseline and 3 months. Authors suggest to analyze their results with caution due to limited number of patients in each subgroups. They speculate that it was probably nonspecific response of the endothelium to the different types of treatment [36]. Considering that sE-selectin is involved in leukocyte and cancer cell extravasation, homing, adhesion, proliferation, cellular dormancy, drug resistance, and tumor progression. It is a potentially promising target for suppression of cancer cell spread into distant tissues [37]. According to Muz et al., as a specific E-selectin antagonist, Uproleselan manipulates the tumor microenvironment by suppression extrinsic and adhesion phases of metastasis. Thus, blocking of E-selectin leads to arrest of tumor dissemination. Additionally, Uproleselan pushes cancer cells to the bloodstream, making them more accessible to chemotherapeutic agents [38].

In our study, we also discovered that patients, despite the treatment pattern, had higher post-treatment sP-selectin concentrations. Adjuvant treatment causes platelet activation which leads to prothrombotic activation. This observation is consistent with Mills et al. who showed higher sP-selectin levels after chemotherapy. P-selectin promotes the generation of platelet—tumor cell complexes in the circulation and stimulates extravasated into a distant site. Authors suggest that chemotherapy lead to enhancement of only platelet activation. Inflammation in response to chemotherapeutic agents is predominantly associated with pre-therapeutic inflammation but also potentially to treatment pattern and baseline tumor biology [39]. This statement is confirmed by our study since we have found positive correlations between pre- and post-treatment concentrations of sP- and sE-selectins (Figure 2).

Moreover, in our investigation, we observed that post-treatment vWF concentrations were higher in patients, regardless of treatment pattern, who were treated with anthracycline chemotherapy. The concentration of vWF is higher after adjuvant treatment because it causes vascular endothelial damage and increases its prothrombotic nature. vWF is considered a marker of vascular endothelial cell damage. Our results corroborate the study by Gil-Bazo et al. who showed increased vWF levels after surgery, which may be due to secondary tissue damage [40]. In their study, Giri et al. observed higher levels of vWF in patients undergoing chemotherapy, which may mean that chemotherapy predisposes patients to develop thrombosis and endothelial dysfunction [41]. Mills et al. also demonstrated a higher vWF concentration after chemotherapy in breast cancer patients. They also link their results with endothelial damage and a higher probability of hypercoagulability state in post-chemotherapy patient [39].

### 4.2. Endothelial Markers’ Relationship with LAR Concentrations before and after Treatment

The next step of the analysis was to demonstrate the relationship between endothelial markers and LAR values. We observed trend towards significance in respect to the higher the post-treatment LAR level and the higher pre-treatment sP-selectin score in breast cancer patients. This may be due to the fact that the higher the LAR, the higher the systemic inflammation. It is well-established that pro-inflammatory state potentiates angiogenic switch and then new vessel network formation and finally tumor cells dissemination [42]. Inflammation has been acknowledged as a ‘red flag of all cancer stages’. Since during this process platelets are activated and secrete numerous compounds, including adhesion molecules (i.e., P-selectin, E-selectin, VCAM-1, ICAM-1), pro-angiogenic, pro-mitogenic (vascular endothelial growth factor). This is responsible for increase vessel permeability and subsequently formation of leukocyte-platelet complexes and the migration of leukocytes across the endothelium [43]. There is strong evidence that P-selectin mRNA levels in mice models are up-regulated by pro-inflammatory cytokines, i.e., TNF-α or IL-1β [44]. Additionally, the soluble form of P-selectin may exhibit proatherogenic and prothrombotic effects. Therefore, it is involved, among other things, in the pathogenesis of thromboembolic complications in breast cancer patients [45]. Ay et al. noted that sP-selectin concentrations were significantly higher among cancer patients with venous thromboembolism (VTE) in respect to their counterparts without VTE [46]. Tumor progression is associated with overexpression of tissue factor the main initiator of coagulation, which is responsible for clotting-dependent induction of angiogenesis [47]. Thus, in metastatic tumor nature both angiogenesis and TF-dependent coagulation processes collaborate as perfect match ‘soulmates’.

### 4.3. Regression Model Analysis of Research Variables as Prognostic Indicators

The following stage of analysis was to test the regression model analysis of the variables as predictors. We performed a linear regression in which the lower post-treatment sP-selectin concentration was correlated with a shorter DFS with a tendency to significance in Model 3, which was adjusted for age, BMI, parity, menopausal status, and smoking status. Likewise, when age, BMI, parity, menopausal status, smoking status, tumor stage, tumor diameter, intrinsic type, histological type, and nodal involvement were all controlled for, the outcome showed a trend towards significance, with a shorter DFS associated with a lower post-treatment sP-selectin concentration. Furthermore, we produced ROC and Kaplan–Meier curves as an additional analysis. We showed that the post-treatment sP-selectin concentration based on the cut-off point according to the ROC curve is a predictor of breast cancer risk in DFS (a result with a trend towards statistical significance) and confirms the result we obtained by performing a linear regression. Furthermore, in our ROC and Kaplan–Meier analysis, we demonstrated that the higher the sP-selectin concentration before treatment, the worse the prognosis. These findings were supported by the Cox regression model, since patients with a pre-treatment sP-selectin higher than 265.05 ng/mL or 247.40 ng/mL (both cut-off points) demonstrate 4.48- and 7.55-fold higher risk of disease recurrence, respectively. Our findings are in line with Ferroni et al., who noted that pre-surgical high sP-selectin might serve as a prognostic marker in the management as well as in predicting recurrence and mortality in colorectal cancer patients [48]. Additionally, Ay et al. suggest that a high sP-selectin concentration is a predictive factor for cancer-dependent VTE [46]. Graf et al. claim that sP-selectin in tumor microenvironment increases platelet mobilization and may provoke cancer progression and metastases. Generally, a high concentration of sP-selectin at cancer diagnosis is associated with more advanced and metastatic tumors [49]. It is worth to emphasize that high pre-treatment levels of sP-selectin prognosticate poorly while its elevated post-treatment levels indicate better future outcomes. There is a lack of data analyzing the prognostic value of post-treatment sP-selectin levels. Therefore, it is difficult to define the cause of this condition, this observation should be confirmed by larger study.

Analyzing sE-selectin in this regard, it showed prognostic value only in Cox regression analysis. Surprisingly, subjects with a pre-treatment sE-selectin concentration lower than 25.04 ng/mL appear to have a higher risk of disease recurrence. However, this observation is inconsistent with previous studies of Ramcharan et al., Mann et al. and Muz et al. [36,37,38]. Since, we should expect opposite results due to the fact that high sE-selectin concentration is associated with a shorter survival rate [50]. Apparently, a low pre-treatment concentration of sE-selectin as a negative biomarker of future outcomes may surprise, but in tumor biology is so many aspects still uncovered, which require further elucidation.

We also conclude that the post-treatment LAR concentration according to the median and cut-off point of the ROC curve is a predictor of breast cancer risk in OS and DFS. ROC curves were used for additional analysis (median and ROC cut-off, respectively) have a 10.32-fold higher risk of disease relapse. This study’s findings revealed that the post-treatment LAR was the best predictor of disease relapse. We also performed Kaplan–Meier curves in which we found that patients with higher LAR levels had worse OS and DFS. Additionally, according to a multivariate and univariate Cox regression model, we showed an increase in the risk of disease relapse with an increase in the post-treatment LAR concentration. Thus, subjects with a pre-treatment LAR concentration higher than 0.46 appear to have a 11.32-fold higher risk of disease recurrence; also, patients with post-treatment LAR concentrations higher than 0.82 and 0.83. Diaz et al. hypothesized in their study that the LAR has an impact on the survival of patients with epithelial ovarian cancer. They performed a Kaplan–Meier survival analysis in which women with a low LAR showed a statistically longer disease-specific survival (57 months) compared to those with a median or high level. However, when they performed a Cox regression analysis, the LAR did not prove to be a statistically significant prognostic factor [51]. Similarly, Słomian et al. demonstrated a significant correlation between the LAR (before treatment) and the effects of treatment, i.e., the lower the ratio, the better the clinical response [25].

Furthermore, a lower pre-treatment vWF concentration is a predictor of a high risk of breast cancer recurrence. This result is confirmed by the Kaplan–Meier curves and multivariate and univariate Cox regression. Terraube et al. achieved similar results in their investigation examining whether vWF is involved in metastatic development. They discovered a substantial increase in the frequency of lung metastatic foci in vWF-null mice compared to wild-type mice in an experimental model. They discovered that greater metastasis was caused by higher survival of tumor cells in the lung during the first 24 h in the absence of vWF [52]. Tigges et al. suggest that low vWF amount can drive the development of new blood vessels around the tumor [53]. However, there are conflicting results since Pepin et al. suggest that high vWF concentration is associated with cancer cell spread [54], which may confirm our results related to a negative prognostic value of a post-treatment vWF concentration. Since patients with higher than 2621.25 mU/mL post-treatment vWF levels present a 6.26-fold higher risk of disease recurrence.

### 4.4. Limitations of This Study

We would like to highlight some of this study’s limitations. We enrolled a small number of patients and recruited them from only one study center, which itself limits the number of patients. The sample size was determined by obtaining patients’ permission to participate and having them meet very strict inclusion criteria. Additionally, the low ethnic diversity among patients might be associated with limited ability to applicate our results to other ethnic population. Similar study in multicenter mode should be designed in order to reach a larger population that enroll most existing races and conditions. As we recruited non-metastatic, I–II stage IBrC patients, we are unable to provide the prognostic value for larger and more advanced tumors.

## 5. Conclusions

Despite the small number of luminal IBrC patients included in this study, our findings reveal a few key points: (1) Regardless of treatment pattern, adjuvant IBrC therapy most likely boosted the LAR and endothelial marker concentrations. (2) Thus, post-treatment endothelial markers are predominantly associated with its pre-therapeutic values but also potentially with treatment pattern and baseline tumor characteristics. (3) The post-treatment LAR levels appear to have been associated with future outcomes in patients with luminal IBrC, as LAR levels higher than 0.82 (median cut-off) and 0.83 (ROC cut-off) have been shown to promote the probability of relapse and mortality in the IBrC cohort. (4) The pre-treatment sP-selectin levels appear to be related to future outcomes in patients with luminal IBrC, with LAR levels more than 265.05 ng/mL (median cut-off) and 247.40 ng/mL (ROC cut-off) increasing the likelihood of recurrence and death in the IBrC cohort. (5) Based on the four linear regression models only the pre-treatment sP-selectin levels showed prognostic value. (6) Higher pre-treatment sP-selectin and post-treatment LAR levels were mainly associated with poorer future outcomes for patients with luminal IBrC.

## Figures and Tables

**Table 1:** Preliminary and clinical characteristics of the study group.

Demographic and Clinical Data	Overall (*n* = 70)	Patients without Progression (*n* = 59)	Patients with Progression (*n* = 11)
		*n* (%)	
Age			
<55 years	35 (50%)	30 (51%)	5 (45.5%)
>55 years	35 (50%)	29 (49%)	6 (54.5%)
Menopausal status			
Pre-menopausal	26 (37%)	22 (37%)	4 (36%)
Post-menopausal	44 (63%)	37 (63%)	7 (64%)
BMI (kg/m^2^)			
Normal (18.5 ≤ 24.99)	34 (48.6%)	27 (46%)	7 (64%)
Overweight (25 ≤ 29.99)	23 (32.8%)	22 (37%)	1 (9%)
Obese (>30)	13 (18.6%)	10 (17%)	3 (27%)
Parity status			
0	6 (9%)	3 (5%)	3 (27.2%)
1–2	50 (71%)	46 (78%)	4 (36.4%)
3 and more	14 (20%)	10 (17%)	4 (36.4%)
Localization of tumor			
Right breast	36 (51%)	31 (53%)	5 (45.5%)
Left breast	34 (49%)	28 (47%)	6 (54.5%)
Lymph node status			
N0	53 (76%)	46 (78%)	7 (64%)
N1	17 (24%)	13 (22%)	4 (36%)
Histological type			
IDC	61 (87%)	51 (86%)	10 (91%)
ILC	9 (13%)	8 (14%)	1 (9%)
TNM staging classification			
T1	48 (69%)	44 (75%)	4 (36%)
T2	22 (31%)	15 (25%)	7 (64%)
Grade according to Elston–Ellis			
1 + 2	61 (87%)	53 (90%)	8 (73%)
3	9 (13%)	6 (10%)	3 (27%)
Molecular type			
Luminal A (HR+/HER2−/Ki-67 < 20%)	50 (71%)	46 (78%)	4 (36%)
Luminal B (HR+/HER2−/Ki-67 ≥ 20%)	16 (23%)	10 (17%)	6 (55%)
Luminal B HER2+ (HR+ HER2+)	4 (6%)	3 (5%)	1 (9%)
Stage			
I	35 (50%)	33 (56%)	2 (18%)
II	35 (50%)	26 (44%)	9 (82%)
Progesterone receptor (PR)			
Negative	5 (7%)	3 (5%)	2 (18%)
Positive	65 (93%)	56 (95%)	9 (82%)
E-cadherin			
Negative	5 (7%)	5 (8%)	0
Positive	65 (93%)	54 (92%)	11 (100%)
Ki-67			
<20%	50 (71%)	45 (76%)	5 (45.5%)
≥20%	20 (29%)	14 (24%)	6 (54.5%)

BMI: body mass index; N0: lack of lymph node metastases; N1: spread to auxiliary lymph nodes; IDC: invasive ductal carcinoma; ILC: invasive lobular carcinoma; T1: tumor diameter < 2 cm; T2: tumor diameter > 2 cm to <5 cm; HR+: hormone receptor positive; HER2−: human epidermal growth factor receptor 2 negative; HER2+: human epidermal growth factor receptor 2 positive; Ki67: marker of proliferation.

**Table 2:** Treatment characteristics of patients in respect of LAR.

Feature/Number of Patients	Pre-Treatment LAR Value	Post-Treatment LAR Value	*p*-Value
Surgery	*p* = 0.2260	*p* = 0.8974	
BCS + Radiotherapy—BCT	0.35	0.83	**0.0192**
56	0.11/0.59	0.33/1.39	
Mastectomy	0.47	0.8	**0.0005**
14	0.21/0.78	0.40/1.35	
Chemotherapy	*p* = 0.1458	*p* = 0.3883	
Anthracycline based	0.28	0.73	**0.0089**
23	0.12/0.60	0.24/1.23	
Non-anthracycline	0.61	1.05	**0.4652**
4	0.15/1.48	0.43/2.17	
No	0.47	0.83	**0.0014**
43	0.21/0.83	0.46/1.35	
Endocrine therapy *	*p* = 0.5473	*p* = 0.1923	
Tamoxifen	0.48	0.71	0.0708
40	0.22/0.79	0.36/1.22	
Inhibitor aromatase	0.4	1.15	**0.0042**
17	0.24/0.56	0.52/1.42	
Tamoxifen and inhibitor aromatase	0.61	1.17	**0.0280**
7	0.18/1.02	0.84/1.93	

Data are expressed as the median (Me) and interquartile range (IQR); *p*-values < 0.05 were considered to indicate statistical significance and are marked in bold, underlined *p*-values represent closeness to statistical significance. BCS: breast-conserving surgery; BCT: breast-conserving therapy. * Due to limited space in the manuscript and lack of the significance patients with other types of hormonal therapy (four cases) and without endocrine therapy (two patients) were removed.

**Table 3:** Treatment characteristics of patients in respect of sE-selectin.

Feature/Number of Patients	Pre-Treatment sE-Selectin Concentration (ng/mL)	Post-Treatment sE-Selectin Concentration (ng/mL)	*p*-Value
Surgery	*p * = 0.0840	*p* = 0.7225	
BCS + Radiotherapy—BCT	35.36	147.23	**<0.0001**
56	29.20/45.60	(59.43)	
Mastectomy	28.59	153.85	**0.0015**
14	21.95/36.51	(62.49)	
Chemotherapy	***p* = 0.0125**	***p* = 0.0081**	
Anthracycline	29.7	128.42	**0.0001**
23	21.72/35.56	77.02/143.96	
Non-anthracycline	37.89	171.61	**0.0679**
4	36.69/51.95	159.55/205.32	
No	35.36	180.58	**<0.0001**
43	28.31/47.40	92.56/202.70	
Endocrine therapy *	*p* = 0.4964	*p* = 0.1460	
Tamoxifen	35.36	133.44	**<0.0001**
40	26.89/45.10	81.38/187.20	
Inhibitor aromatase	32.5	165.55	**0.0003**
17	30.20/62.50	135.93/224.24	
Tamoxifen and inhibitor aromatase	33.79	179.26	**0.0180**
7	21.54/38.90	128.42/229.48	

Data are expressed as the median (Me) and interquartile range (IQR) or means ± standard deviation; *p*-values < 0.05 were considered to indicate statistical significance and are marked in bold, underlined *p*-values represent closeness to statistical significance. BCS: breast-conserving surgery; BCT: breast-conserving therapy. * Due to limited space in the manuscript and lack of the significance patients with other types of hormonal therapy (4 cases) and without endocrine therapy (2 patients) were removed.

**Table 4:** Treatment characteristics of patients in respect of sP-selectin.

Feature/Number of Patients	Pre-Treatment sP-Selectin Concentration (ng/mL)	Post-Treatment sP-Selectin Concentration (ng/mL)	*p*-Value
Surgery	*p* = 0.3709	*p* = 0.4295	
BCS + Radiotherapy—BCT	253.95	1687.86	**<0.0001**
56	190.40/344.10	1051.14/2447.81	
Mastectomy	342.5	2071.25	**0.0015**
14	184.30/383.25	1617.68/2508.57	
Chemotherapy	*p* = 0.4316	*p* = 0.9237	
Anthracycline	266.95	1864.48	**0.0001**
23	177.5/344.10	(781.04)	
Non-anthracycline	299.3	1607.3	**0.0679**
4	188.63/439.60	(739.12)	
No	263.15	1886.39	**<0.0001**
43	193.05/383.25	(881.77)	
Endocrine therapy *	*p* = 0.2307	***p* = 0.0015**	
Tamoxifen	286	1859.1	**<0.0001**
40	197.65/380.35	(820.91)	
Inhibitor aromatase	276.65	1947.98	**0.0003**
17	192.15/359.40	(965.53)	
Tamoxifen and inhibitor aromatase	247.4	2055.24	**0.0180**
7	177.50/344.10	(729.06)	

Data are expressed as the median (Me) and interquartile range (IQR) or means ± standard deviation; *p*-values < 0.05 were considered to indicate statistical significance and are marked in bold. BCS: breast-conserving surgery; BCT: breast-conserving therapy. * Due to limited space in the manuscript and lack of the significance patients with other types of hormonal therapy (four cases) and without endocrine therapy (two patients) were removed.

**Table 5:** vWF-related patient treatment characteristics.

Feature/Number of Patients	Pre-Treatment vWF Concentration (mU/mL)	Post-Treatment vWF Concentration (mU/mL)	*p*-Value
Surgery	*p* = 0.9095	*p* = 0.2041	
BCS + Radiotherapy—BCT	575.61	2254.19	**<0.0001**
56	(240.3)	1473.88/2928.03	
Mastectomy	567.21	1633.26	**0.0012**
14	(270.85)	1299.04/2730.81	
Chemotherapy	*p* = 0.6517	***p* = 0.0486**	
Anthracycline	582.5	2579.4	**<0.0001**
23	(219.47)	1902.00/3242.13	
Non-anthracycline	745.85	1665.7	**0.0679**
4	(140.01)	1578.01/1950.54	
No	553.2	1802.17	**<0.0001**
43	(260.08)	1290.09/2798.43	
Endocrine therapy *	*p* = 0.3784	*p* = 0.5300	
Tamoxifen	569.9	2038.18	**<0.0001**
40	439.00/737.70	1290.09/2865.13	
Inhibitor aromatase	700	1873.39	**0.0003**
17	500.00/811.80	1564.76/2898.46	
Tamoxifen and inhibitor aromatase	569.9	2392.3	**0.0180**
7	111.00/600.00	920.97/3919.44	

Data are expressed as the median (Me) and interquartile range (IQR) or means ± standard deviation; *p*-values < 0.05 were considered to indicate statistical significance and are marked in bold. BCS: breast-conserving surgery; BCT: breast-conserving therapy. * Due to limited space in the manuscript and lack of the significance patients with other types of hormonal therapy (four cases) and without endocrine therapy (two patients) were removed.

**Table 6:** Endothelial markers according to pre-treatment LAR values.

	Pre-Treatment LAR Low Value (<0.27) *n* = 23	Pre-Treatment LAR Moderate Value (0.27–0.65) *n* = 25	Pre-Treatment LAR High Value (>0.65) *n* = 22	*p*-Value
Pre-Treatment	29.76	32.91	40.95	0.1391
sE-selectin Concentration (ng/mL)	25.25/36.87	27.61/48.05	32.11/46.00	
Post-Treatment	131.55	165.17	179.26	0.2829
sE-selectin Concentration (ng/mL)	81.13/172.55	84.31/224.24	116.55/192.51	
Pre-Treatment	252.1	308.08	247.4	0.532
sP-selectin Concentration (ng/mL)	177.50/327.95	215.75/378.08	210.60/383.25	
Post-Treatment	1916.87	1941.61	1870.24	0.9607
sP-selectin Concentration (ng/mL)	(821.54)	(821.33)	(866.22)	
Pre-Treatment vWF	553.23	538.06	653.55	0.2312
Concentration (mU/mL)	(224.74)	(246.07)	(246.01)	
Post-Treatment vWF	2098.39	2123.11	2261.09	0.8478
Concentration (mU/mL)	(816.75)	(1168.85)	(960.44)	

LAR: leptin-to-adiponectin ratio; vWF: von Willebrand factor.

**Table 7:** Endothelial markers according to post-treatment LAR values.

	Post-Treatment LAR Low Value (<0.60) *n* = 25	Post-Treatment LAR Moderate Value (0.60–1.04) *n* = 19	Post-Treatment LAR High Value (>1.04) *n* = 26	*p*-Value
Pre-Treatment	32.38	38.76	32.5	0.4678
sE-selectin Concentration (ng/mL)	25.67/47.30	28.76/45.64	25.25/39.96	
Post-Treatment	137.19	153.89	155.77	0.5101
sE-selectin Concentration (ng/mL)	(64.26)	(58.67)	(56.28)	
Pre-Treatment	242.35	249.38	327.95	0.0528
sP-selectin Concentration (ng/mL)	158.35/318.00	194.03/310.78	237.75/380.35	
Post-Treatment	1597.57	2097.78	1687.86	0.6921
sP-selectin Concentration (ng/mL)	986.10/2500.01	1513.27/2444.38	1224.87/2550.92	
Pre-Treatment vWF	673.95	500	569.9	0.1947
Concentration (mU/mL)	461.00/773.35	400.00/647.90	382.10/811.80	
Post-Treatment vWF	2456.8	2101.08	2148.9	0.8757
Concentration (mU/mL)	1532.14/3178.53	1581.64/2617.41	1449.11/2949.19	

Underlined *p*-value represent closeness to statistical significance; LAR: leptin-to-adiponectin ratio; vWF: von Willebrand factor.

**Table 8:** Linear regression models for disease-free survival predictors in breast cancer patients.

		Model 1	Model 2	Model 3	Model 4
Pre-Treatment LAR value	Beta	0.0374	−0.0181	−0.0202	−0.0009
	*p*-value	0.7656	0.9144	0.9042	0.9949
Post-Treatment LAR value	Beta	−0.0331	−0.0831	0.0073	−0.0835
	*p*-value	0.7972	0.5616	0.9602	0.5151
Pre-Treatment sE-selectin	Beta	0.0839	0.054	0.1127	0.0619
Concentration (ng/mL)	*p*-value	0.5023	0.6929	0.4046	0.6048
Post-Treatment sE-selectin	Beta	0.0748	0.0539	0.0283	0.0742
Concentration (ng/mL)	*p*-value	0.5627	0.6864	0.8324	0.5313
Pre-Treatment sP-selectin	Beta	0.0213	0.0525	0.0306	0.0171
Concentration (ng/mL)	*p*-value	0.8635	0.6928	0.8187	0.8831
Post-Treatment sP-selectin	Beta	−0.1619	−0.1566	−0.2576	−0.2437
Concentration (ng/mL)	*p*-value	0.2076	0.2357	0.0504	0.0583
Pre-Treatment vWF	Beta	0.1682	0.1496	0.1186	0.0641
Concentration (mU/mL)	*p*-value	0.1651	0.2377	0.3519	0.5632
Post-Treatment vWF	Beta	−0.0292	−0.0236	−0.0406	−0.0551
Concentration (mU/mL)	*p*-value	0.8125	0.8519	0.7476	0.6252

Model 1 adjusted for age. Model 2 adjusted for age, BMI, parity, and menopausal status. Model 3 adjusted for age, BMI, parity, menopausal status, and smoking status. Model 4 adjusted for age, BMI, parity, menopausal status, smoking status, tumor stage, tumor diameters, intrinsic type, histological type, and nodal involvement. Underlined *p*-values represent closeness to statistical significance.

## Data Availability

The data presented in this study are available in this article.

## Supplementary Materials

The following supporting information can be downloaded at: https://www.mdpi.com/article/10.3390/biomedicines11082246/s1, Figure S1: Kaplan-Meier curves for the overall survival (OS) and disease-free survival (DFS) analysis regarding (A,B) pre-treatment LAR value according to median value cut- off; (C,D) pre-treatment LAR concentration according to ROC cut-off; Figure S2: Kaplan-Meier curves for the overall survival (OS) and disease-free survival (DFS) analysis regarding (A,B) pre-treatment sE-selectin concentration according to median value cut- off; (C,D) pre-treatment sE-selectin concentration according to ROC cut-off; Figure S3: Kaplan-Meier curves for the overall survival (OS) and disease-free survival (DFS) analysis regarding (A,B) post-treatment sE-selectin concentration according to median value cut- off; (C,D) post-treatment sE-selectin concentration according to ROC cut-off; Figure S4: Kaplan-Meier curves for the overall survival (OS) and disease-free survival (DFS) analysis regarding (A,B) post-treatment vWF concentration according to median value cut- off; (C,D) post-treatment vWF concentration according to ROC cut-off; Table S1: Predictive accuracy results for LAR and endothelial markers before treatment; Table S2: Calculated median and ROC cut-off point values of investigated parameters before treatment; Table S3: Predictive accuracy results for LAR and selectins after treatment; Table S4: Calculated median and ROC cut-off point values of investigated parameters after treatment.
