# Supplementary material for: Complex Analysis of Endothelial Markers as Potential Prognostic Indicators in Luminal Invasive Breast Carcinoma Patients: Outcomes of a Six-Year Observational Study
Source: Biomedicines. 2023 Aug 10;11(8):2246. doi: 10.3390/biomedicines11082246 (PMC10452676; doi:10.3390/biomedicines11082246)
Supplement: Supplementary file 1 [file biomedicines-11-02246-s001.zip › biomedicines-2446607-SI.pdf]

SUPPLEMENTARY MATERIALS

1. Receiver Operating Characteristic (ROC) analysis of parameters before treatment.

Table S1 shows the ROC data determined to estimate the prognostic value of the concentrations of the examined parameters before treatment for predicting the OS and PFS. The areas under the curve with 95% confidence intervals (AUC, 95% thresholds with sensitivity and specificity) were established.

According to the study, the best predictor of relapse was found to be the pre-treatment vWF concentration with close to statistical significance (AUC = 0.642, p = 0.0870). A pre-treatment vWF concentration of 600 mU/ml with a sensitivity of 88.9% and a specificity of 47.5% using the maximum value of the Youden index was considered the best cut-off value to distinguish patients with or without relapse.

Table S1. Predictive accuracy results for LAR and endothelial markers before treatment.

ROC data	Destimulant	Destimulant	Stimulant	Destimulant
	**Pre-Treatment LAR Value ng/mL**	**Pre-Treatment sE-selectin Concentration ng/mL**	**Pre-Treatment sP-selectin Concentration ng/mL**	**Pre-Treatment vWF Concentration mU/mL**
AUC	0.544	0.586	0.612	0.642
Youden index	0.25	0.27	0.36	0.36
Cut-off point	0.59	25.04	247.40	600.00
Sensitivity (%)	88.9	44.4	88.9	88.9
Specificity (%)	36.2	82.8	47.5	47.5
Positive predictive value (%)	17.8	28.6	20.5	20.0
Negative predictive value (%)	95.5	90.6	96.6	96.7
Accuracy (%)	43.3	77.6	52.9	52.9
p-value	0.6221	0.4365	0.1315	<u>0.0870</u>

Underlined *p*-values represent closeness to statistical significance.

2. Survival Analysis Regarding Pre-Treatment LAR and endothelial markers

Table S2. Calculated median and ROC cut-off point values of investigated parameters before treatment.

	**Pre-Treatment LAR Value ng/mL**	**Pre-Treatment sE-selectin Concentration ng/mL**	**Pre-Treatment sP-selectin Concentration ng/mL**	**Pre-treatment vWF Concentration mU/mL**
Medians	0.46	33.79	265.05	569.90
ROC cut-off points	0.59	25.04	247.40	600.00

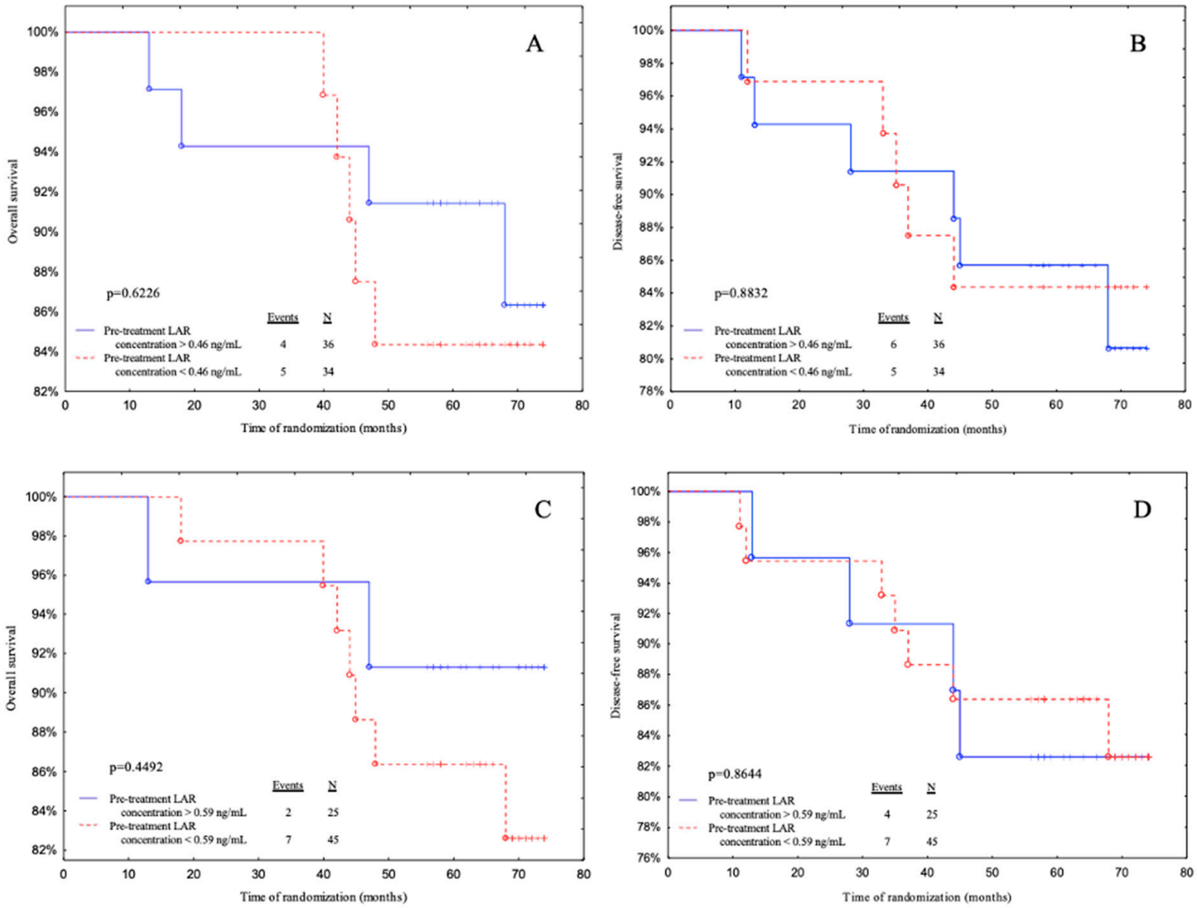

Figure S1. Kaplan-Meier curves for the overall survival (OS) and disease-free survival (DFS) analysis regarding (**A**,**B**) pre-treatment LAR value according to median value cut- off; (**C**,**D**) pre-treatment LAR concentration according to ROC cut-off.

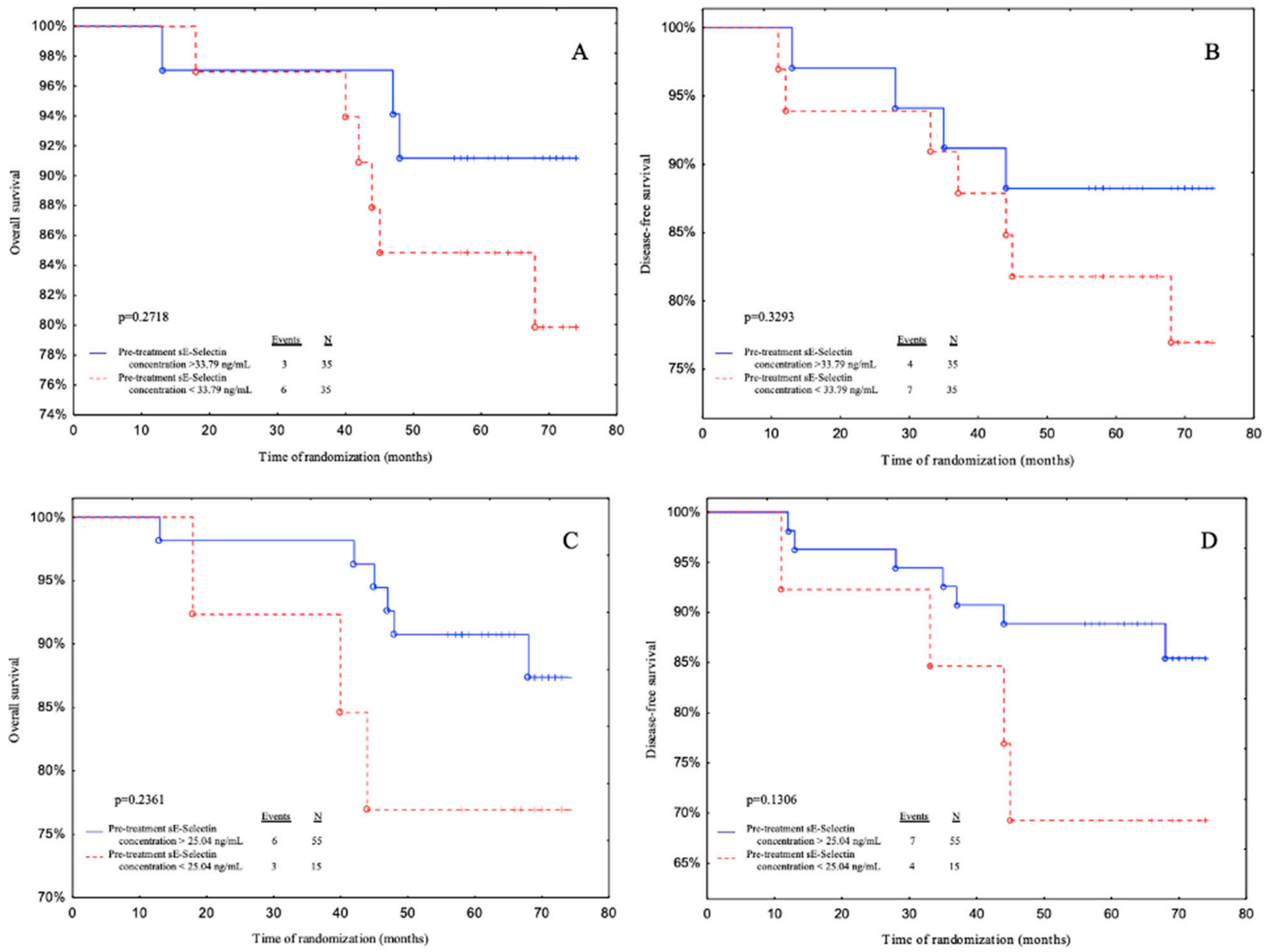

Figure S2. Kaplan-Meier curves for the overall survival (OS) and disease-free survival (DFS) analysis regarding (**A**,**B**) pre-treatment sE-selectin concentration according to median value cut- off; (**C**,**D**) pre-treatment sE-selectin concentration according to ROC cut-off.

3. Receiver Operating Characteristic (ROC) analysis of parameters after treatment.

Table S3 displays the ROC data used to estimate the prognostic value of the examined parameters' concentrations prior to treatment for predicting OS and PFS. The areas under the curve (AUC) with 95% confidence intervals (sensitivity and specificity) were calculated.

According to the study's findings, the post-treatment leptin-to-adiponectin ratio was found to be the best predictor of disease relapse (AUC = 0.741, p = 0.0013). Using the maximum value of the Youden index, the best cut-off value to distinguish patients with or without disease recurrence was identified as a post-treatment LAR of 0.83 ng/mL with sensitivity of 88.9% and specificity of 57.9%.

Table S3. Predictive accuracy results for LAR and selectins after treatment.   .

	**Stimulant**	**Destimulant**	**Stimulant**	**Stimulant**
**ROC data**	**Post-Treatment LAR Value ng/mL**	**Post-Treatment sE-selectin Concentration ng/mL**	**Post-Treatment sP-selectin Concentration ng/mL**	**Post-Treatment vWF Concentration mU/mL**
AUC	0.741	0.505	0.614	0.526
Youden index	0.47	0.18	0.32	0.21

Cut-off point	0.83	153.71	2224.44	2621.25
Sensitivity (%)	88.9	66.7	66.7	55.6
Specificity (%)	57.9	50.9	64.9	65.6
Positive predictive value (%)	25.0	17.6	23.1	19.2
Negative predictive value (%)	97.1	90.6	92.5	90.9
Accuracy (%)	62.1	53.0	65.2	64.3
p-value	**0.0013**	0.9639	0.1981	0.8083

Significant differences are denoted by bold *p*-values.

4. Post-treatment LAR and endothelial markers survival analysis

In the statistical analysis, the cut-off points from the ROC curve for LAR and endothelial markers after treatment were used, and the cut-off point based on the median was calculated (Table S4). The patients were then divided into two groups: those who were below the cut-off point and those who were above it. The OS and DFS of each group were assessed. The median length of follow-up was 68.5 months (interquartile range: 59-72 months). During the study, ten patients died from systemic metastatic disease, with only one recurrence. The rate of recurrence was 15.71 percent.

Table S4. Calculated median and ROC cut-off point values of investigated parameters after treatment.

	**Post-Treatment LAR Value ng/mL**	**Post-Treatment sE-selectin Concentration ng/mL**	**Post-Treatment sP-selectin Concentration ng/mL**	**Post-treatment vWF Concentration mU/mL**
Medians	0.82	152.39	1738.76	2124.99
ROC cut-off points	0.83	153.71	2224.44	2621.25

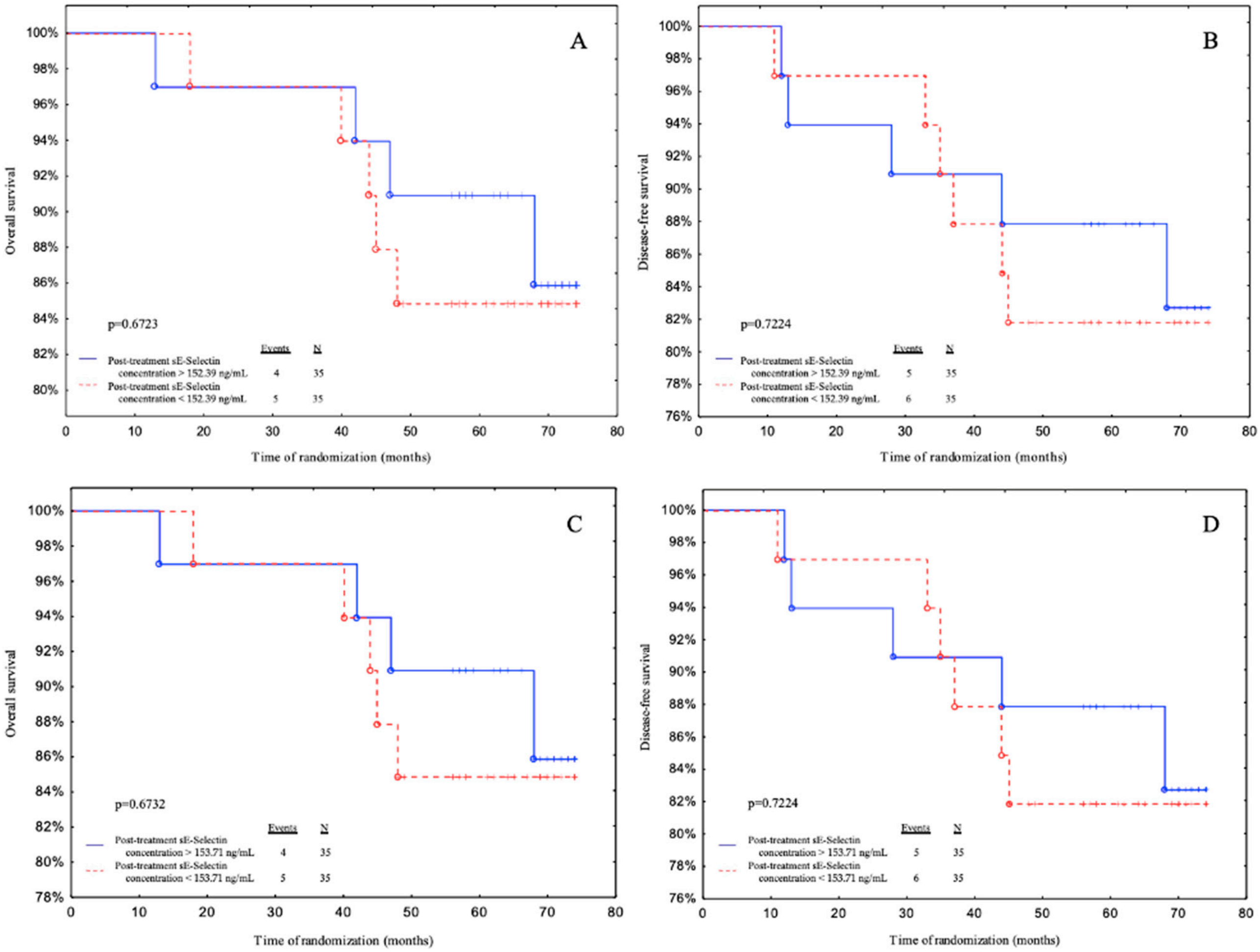

Figure S3. Kaplan-Meier curves for the overall survival (OS) and disease-free survival (DFS) analysis regarding (**A**,**B**) post-treatment sE-selectin concentration according to median value cut- off; (**C**,**D**) post-treatment sE-selectin concentration according to ROC cut-off.

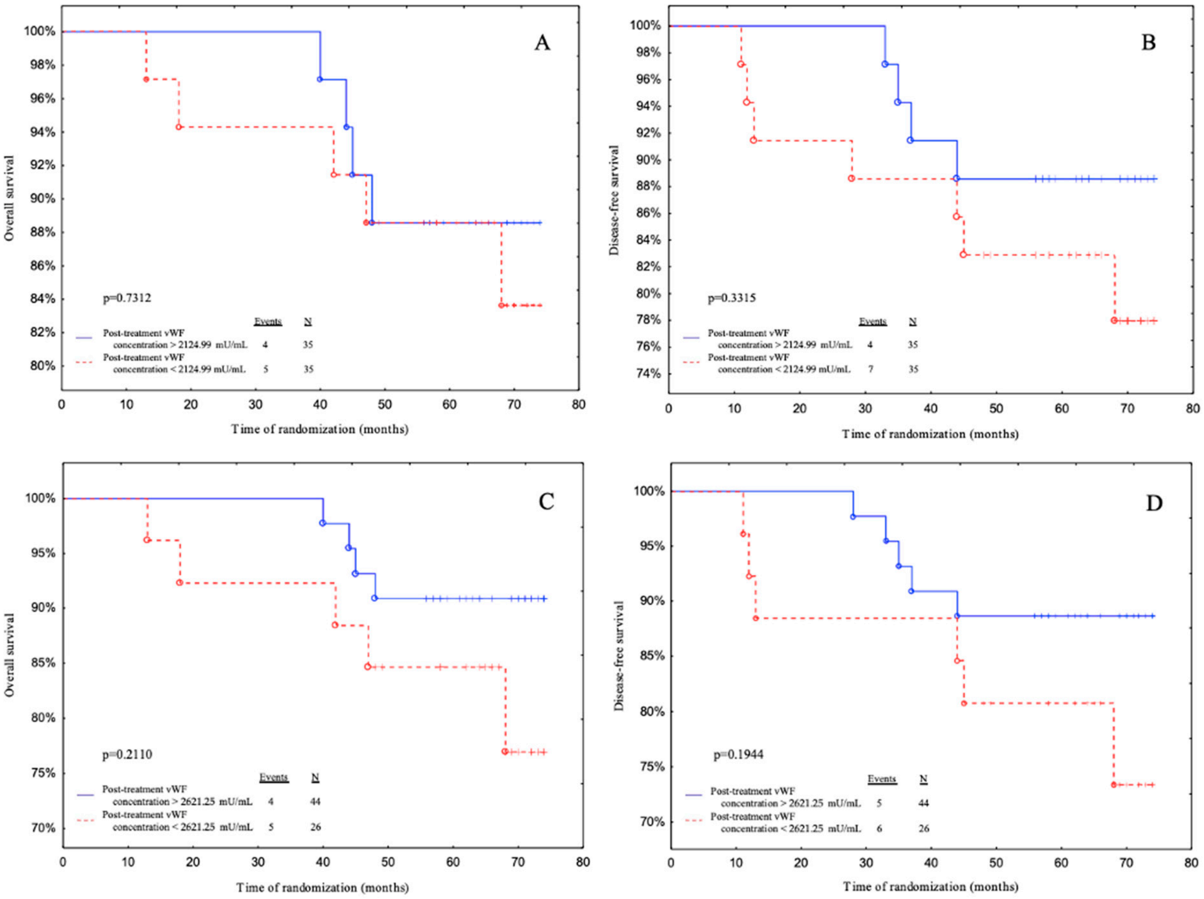

Figure S4. Kaplan-Meier curves for the overall survival (OS) and disease-free survival (DFS) analysis regarding (**A**,**B**) post-treatment vWF concentration according to median value cut- off; (**C**,**D**) post-treatment vWF concentration according to ROC cut-off.
